# Do Ants Need to Estimate the Geometrical Properties of Trail Bifurcations to Find an Efficient Route? A Swarm Robotics Test Bed

**DOI:** 10.1371/journal.pcbi.1002903

**Published:** 2013-03-28

**Authors:** Simon Garnier, Maud Combe, Christian Jost, Guy Theraulaz

**Affiliations:** 1 Centre de Recherche sur la Cognition Animale, UMR-CNRS 5169, Université Paul Sabatier, Bât 4R3, Toulouse, France; 2 CNRS, Centre de Recherches sur la Cognition Animale, Toulouse, France; 3 Department of Biological Sciences, New Jersey Institute of Technology, Newark, New Jersey, United States of America; Ecole Polytechnique Fe´de´rale de Lausanne EPFL, Switzerland

## Abstract

Interactions between individuals and the structure of their environment play a crucial role in shaping self-organized collective behaviors. Recent studies have shown that ants crossing asymmetrical bifurcations in a network of galleries tend to follow the branch that deviates the least from their incoming direction. At the collective level, the combination of this tendency and the pheromone-based recruitment results in a greater likelihood of selecting the shortest path between the colony's nest and a food source in a network containing asymmetrical bifurcations. It was not clear however what the origin of this behavioral bias is. Here we propose that it results from a simple interaction between the behavior of the ants and the geometry of the network, and that it does not require the ability to measure the angle of the bifurcation. We tested this hypothesis using groups of ant-like robots whose perceptual and cognitive abilities can be fully specified. We programmed them only to lay down and follow light trails, avoid obstacles and move according to a correlated random walk, but not to use more sophisticated orientation methods. We recorded the behavior of the robots in networks of galleries presenting either only symmetrical bifurcations or a combination of symmetrical and asymmetrical bifurcations. Individual robots displayed the same pattern of branch choice as individual ants when crossing a bifurcation, suggesting that ants do not actually measure the geometry of the bifurcations when travelling along a pheromone trail. Finally at the collective level, the group of robots was more likely to select one of the possible shorter paths between two designated areas when moving in an asymmetrical network, as observed in ants. This study reveals the importance of the shape of trail networks for foraging in ants and emphasizes the underestimated role of the geometrical properties of transportation networks in general.

## Author Summary

Most ant species form transportation networks, be they foraging trails linking food sources to the main colony or underground galleries connecting the different parts of the nest. As for human transportation networks (roads, airlines, etc.), the design and the placement of the connecting points (or nodes) dramatically affects the movement of individuals and hence the exchanges of material and information. In a previous study, we have shown that the geometrical configuration of these nodes (i.e., the angles between the different exiting branches) can affect the route followed by an ant in a network of galleries and, as a consequence, the efficiency of the pheromone-based recruitment toward a food source. Here we show that we can reproduce these results using ant-like robots with minimal perceptual and cognitive capabilities. We demonstrate that the simple interaction between the displacement of an ant and the geometrical configuration of the gallery network can greatly affect the foraging performances of the colony. This result increases our understanding of how workers move through structures built by ant colonies and more generally points toward possible improvements for the design of man-made transportation networks.

## Introduction

Various ant species build networks of trails that link together nesting sites and exploited resources [1]. These networks are generally formed by one or several dendritic trees originating from the nest of the colony. They can stretch over large distances and display very intricate patterns. For instance, the harvester ant *Messor barbarus* forms trails that persist over several consecutive days and can extend up to 25 meters from the nest entrance [2]. The wood ant *Formica aquilonia*, whose body length is just 5–6 millimeters, can form networks where trails reach 200 meters in length, with up to nine successive branching points per trail [3]. As a last example, the trail system in a colony of leafcutter ants *Atta colombica* can cover an area larger than 1 hectare, with trails extending up to 250 meters from the nest [4].

One major challenge for ant workers is to orient themselves inside such labyrinths and in particular to keep track of the direction of their nest. To do so, they use at least four different, but non-exclusive, types of information. First, they can rely on visual information. Some species use forest canopy [5] or sun position [6] to estimate the direction toward their nest. Others memorize environmental landmarks along their path [7]. Second, they can also use proprioceptive information. Certain ant species approximate the direction toward their nest by summing their successive vectors of movements, measured as step numbers and body rotations [8]–[10]. Third, they can exploit social information, such as the food load of encountered workers. In ants carrying their food (such as seeds or leaf fragments) on surface trails, the proportion of laden ants is higher in the returning flow. Some ants use this difference to correctly reorient themselves on a trail [11].

The last type of information that ants can use to find the direction of their nest lies in the structure of the trail network itself. In several ant species, these networks display a particular property: the mean angle between trails as they branch out symmetrically from the nest lies around 60°, in the range 50°–100° depending on the species (*Leptogenys processionalis* [12]; *Atta sexdens*, *A. capiguara*, *A. laevigata* and *Messor Barbarus* [13]; *Monomorium pharaonis* [14]; *Formica aquilonia* [3]; *Linepithema humile*, unpublished data). Therefore, an ant exiting the nest and moving toward the periphery of the network generally faces symmetrical bifurcations, *i.e.* the two trails that follow a bifurcation deviate by approximately 30° from the original direction of the ant. Conversely, an ant coming back to its nest faces asymmetrical bifurcations: the trail heading toward the nest after a bifurcation deviates less (∼30°) from the ant's original direction than the other trail (∼120°) which leads away from the nest. In this last situation, and in absence of any other information, ants preferentially follow the least deviating trail, as demonstrated in our recent study with the Argentine ant *L. humile* [15], [16]. We also showed that this behavioral bias, associated with the pheromone recruitment of this ant species, led to a significant improvement of the colony's ability to select the shortest route between its nest and a newly discovered food source [16] and depends critically on the branching angle [17].

A question that remains to be elucidated is whether ants reaching a bifurcation actually use its geometry as an orientation cue to decide which trail to follow next, or whether their individual and collective behaviors are in fact the product of a passive interaction with the geometrical structure of the trail network. The answer to this question depends on, for the moment, rare behavioral observations whose conclusions differ according to the experimental procedure and species studied [14], [15].

In order to gain new insight into the role of the trail geometry, we studied the behavior of robotics models of ants, whose perception abilities are known and whose behaviors can be specified. During the last fifteen years, the use of robots to investigate animal behavior has been increasingly popular (see [18]–[20] for a review and examples) and has led to the development of innovative control algorithms [21], [22]. Several attempts have been made to produce ant-like robots that are able to lay and follow pheromone-like trails using heat trails [23], chemical trails [24], glow paint trails [25], virtual trails [26] or light trails [27], [28]. Such trail systems are a promising way of guiding and organizing the activities of robotics swarms in space, particularly in unknown environments. From a biological point of view, these robotic models also offer the possibility of investigating questions related to the influence of the perceptual/cognitive abilities of individual ants on the collective behavior of the colony.

Here we present the results of an experiment where a group of ant-like robots had to establish a route between a starting area and a target area in a network of corridors, mimicking the experiments we performed with ants in our previous studies [15], [16]. For technical convenience pheromone trails were replaced by light trails projected along the paths followed by the robots by a video projector (as proposed in [27], [29] and implemented in [28]). Robots can detect and follow these light trails thanks to two photoreceptors that mimic the antennae of the ants. The robots were tested in two types of networks, one type made only of symmetrical bifurcations and the other type containing asymmetrical bifurcations, as in natural ant networks. Their behavior was kept as minimal as possible to observe just the interaction between the displacement of the ants, their trail laying/following behavior and the structure of the environment. In particular and in contrast to previous simulation work [16], [17], they were not given the capability to measure the angle between the corridors when reaching a bifurcation and therefore they could not make a change of direction based on this information. A comparison between the behavior of the robots and the behavior of ants in our previous experiments demonstrates that simple individual behavioral rules are sufficient to explain the efficient pattern of network exploitation observed in ants. It also helps us to better understand how the physical structure of the environment can affect individual and collective activities in social insects.

## Materials and Methods

### Experimental setup

**Figure 1:**
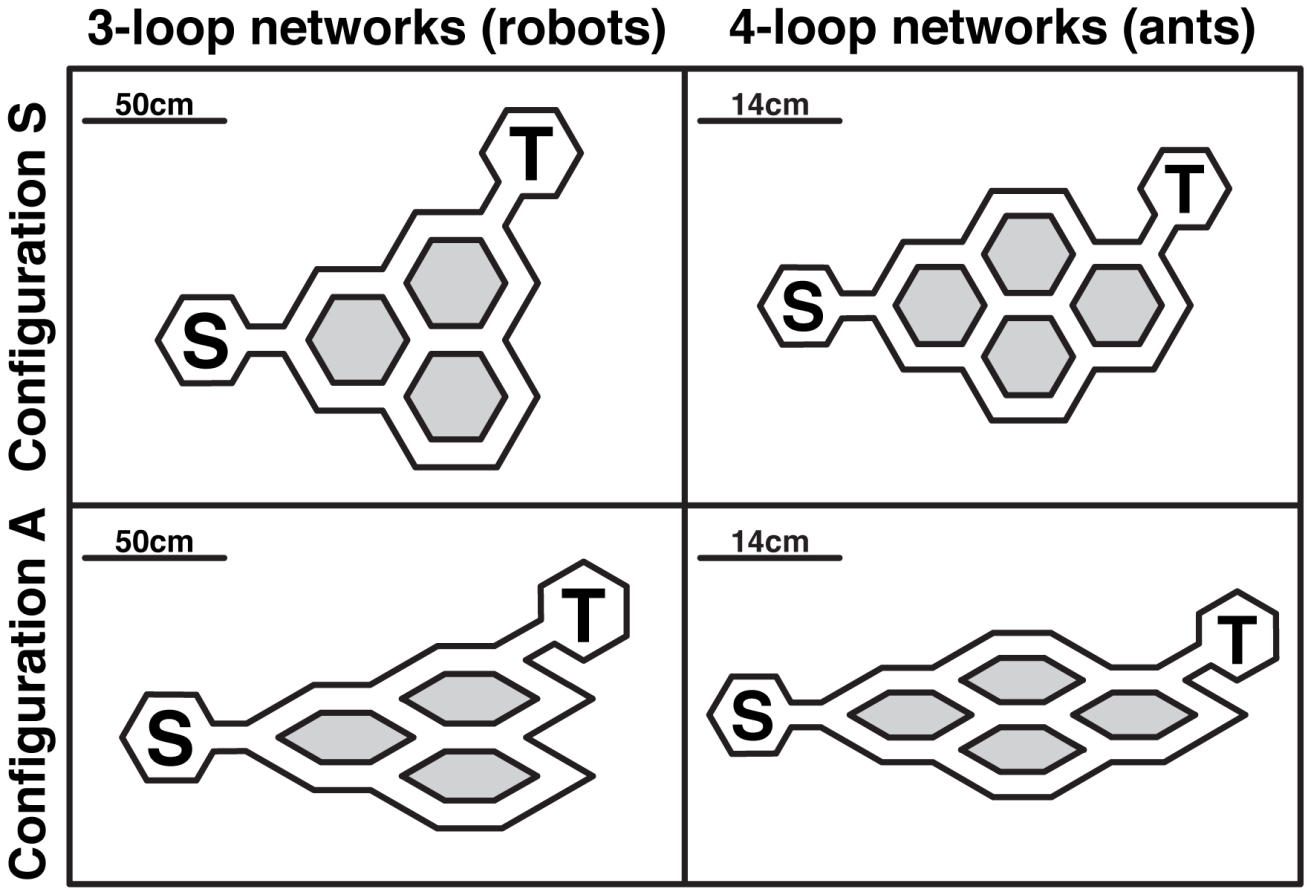
Schematic description of the experimental networks. The left column corresponds to three-loop networks used in our robotic experiments, the right column to four-loop networks used in ant experiments in [16]. The top row corresponds to the symmetrical (S) configuration of each network, the bottom row to their asymmetrical (A) configuration. S marks the starting area, T the target area.

The experimental setup was a scaled-up, simplified version of the setup used in [16] to study the behavior of Argentine ants. The behavior of the ant workers was tested in a maze of corridors carved in a PVC (polyvinyl chloride) board (5 mm wide, about 4–5 times the width of an ant). These corridors mimicked permanent trails that are found in ant species that remove vegetation and debris to form physical routes toward long-lasting food sources [1], [30]. The experimental setup used with the robots was a network of corridors (9 cm width, 4.5 times the width of a robot) built with white cardboard (5 mm thick, wall height of 2.5 cm). In ants, the network was made of four interconnected diamond-shaped loops connecting a starting area (corresponding to the nest of ants) on one end and a target area (corresponding to a food source for instance) on the other end. In robots, the network was made of only three interconnected diamond-shaped loops (see Fig. 1) in order to keep its dimensions within the space allowed by the pheromone deposit device (140×105 cm) while scaling up the length of the diamond-shaped loops by four (robots move 4 times faster than the ants). The starting and target areas were hexagons of the same dimensions (22.5 cm diameter). In this network there were 7 (*vs* 14 for the ants) possible paths of different lengths (shorter path: 86 cm; longer path: 178 cm) that robots could use to go from the starting area to the target area, without using the same segment of the network twice (a corridor between two bifurcations).

Two network configurations were used. In configuration S (for “symmetrical”), each diamond-shaped loop of the network was perfectly symmetrical. As a consequence, all bifurcations of the network were also symmetrical: whatever incoming branch was at a bifurcation, the two other branches deviated by an angle of 60° on the left or on the right. In configuration A (for “asymmetrical”), each diamond-shaped loop of the network was flattened along one of its axes (the same for each loop). As a consequence, the network bifurcations were not always symmetrical anymore: depending on the incoming branch at a bifurcation, the two other branches both deviated by an angle of 30° on the left or on the right (symmetrical side of the bifurcation), or one branch deviated by an angle of 30° in one direction while the other branch deviated by an angle of 120° in the other direction (asymmetrical side of the bifurcation). Except for this difference in the geometry of the bifurcations, configurations S and A were identical: they presented the same topology, had segments of the same length and had the same total length. 15 experimental replicates with 10 robots were performed with each network configuration. Each experimental replicate lasted 60 minutes.

### Robots

**Figure 2:**
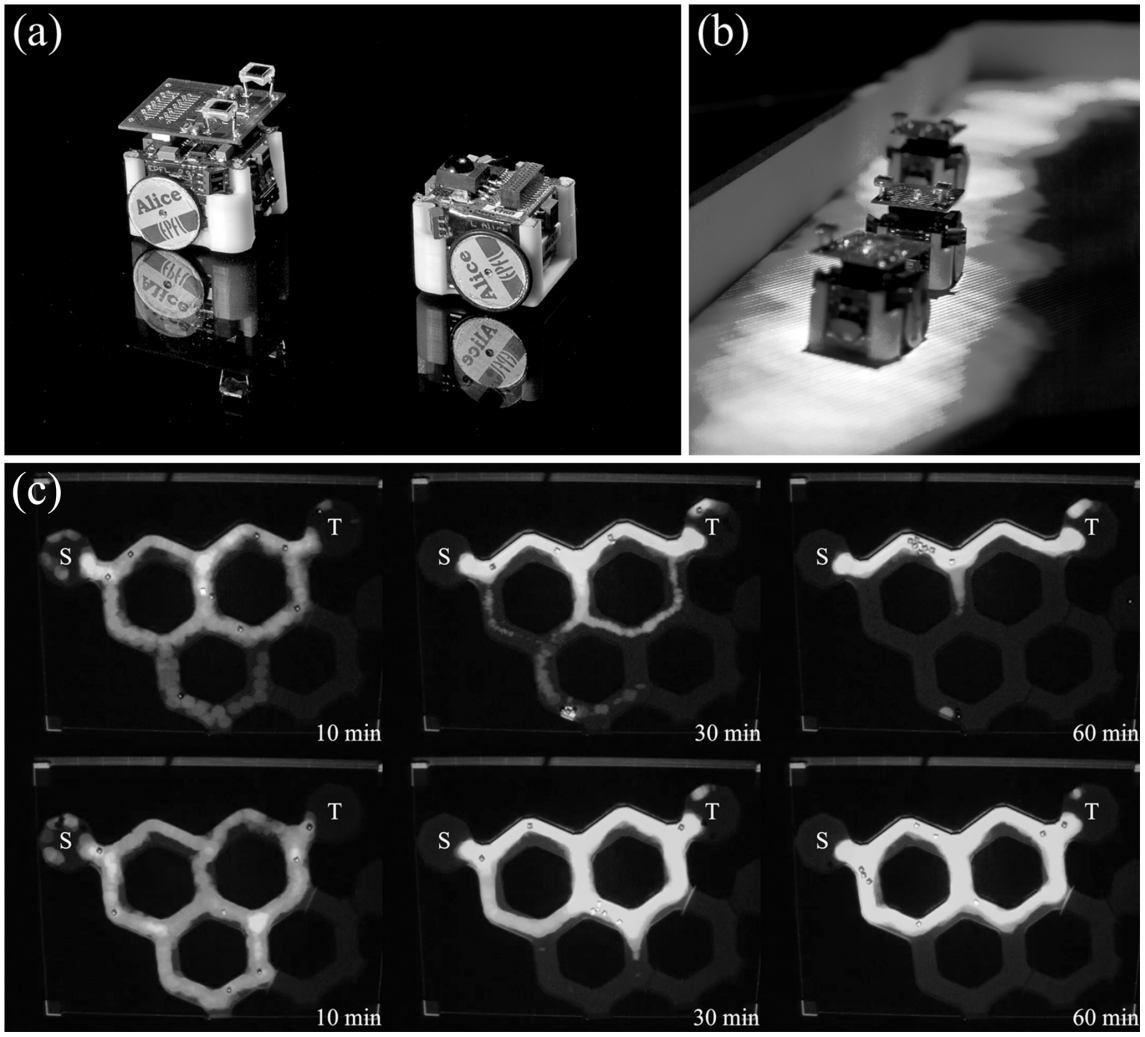
Pictures of the experimental setup. (a) Two Alice robots facing each other, with (left) and without (right) the additional module for light detection. (b) Three Alice robots pursuing a light trail. (c) Typical time course of an experiment with three loops (access to a fourth loop that is visible had been blocked) and symmetrical bifurcations. The letter S indicates the starting area of the network where the robots are placed at the beginning of the experiment. The letter T indicates the target area. The top three pictures represent 3 snapshots of an experiment where a group of 10 robots selects the shortest path.

The micro-robots Alice (see Fig. 2) were designed at the EPFL (Lausanne, Switzerland [31]). They were very small robots (22 mm×21 mm×20 mm) equipped with two watch motors with wheels and tires, with a maximum speed of 40 mm s^−1^. Four infrared (IR) sensors and transmitters were used for detection of the starting and target areas, and for obstacle detection. The front left and front right sensors were oriented 45° toward the left and the right of the robots' moving direction respectively; the front and back sensors were oriented directly ahead and behind of the robots' moving direction respectively. Obstacles could be detected at a maximum distance of 3 centimeters [31]. An add-on module equipped with two photodiodes on each side of the robot and pointing upwards allowed the detection of light gradients. It also carried a red LED (Light Emitting Diode) to permit an easy and reliable tracking in conditions of changing background brightness. A NiMH rechargeable battery provided energy for about 3.5 hours in our experimental conditions. The robots had a microcontroller PIC16LF877 with 8K Flash EPROM memory, 368 bytes RAM and no built-in float operations. Programming was done with the IDE of the CCS-C compiler, and the compiled programs were downloaded in the Alice memory with the PIC-downloader software (EHL elektronika).

### Pheromone deposit device

A firewire digital video camera Unibrain Fire-i400 (resolution 640×480 pixels) was hung about 1.5 m above the robots. It transmitted videos to a Dell Latitude D810 laptop computer via a 1394a PCMCIA card. Image acquisition on the computer was done with the open source CMU 1394 Digital Camera Driver (Robotics Institute, Carnegie Mellon University) and image treatment was done with the open source OpenCV library (Intel Corporation). RGB (Red Green Blue) images were converted into HSV (Hue Saturation Value) space. The rest of the treatment was done on the H channel of the HSV space. This allowed the isolation of a given color in the images (here the red LED carried by robots) regardless of its intensity. White noise was removed thanks to a morphological opening (erosion followed by dilatation) with a 3×3 matrix [32]. The images were then converted to binary images by applying a min-max threshold to isolate the red portion of the H channel. The resulting blobs of pixels were fitted with an ellipse function whose center position provided the position of each robot. Positions were corrected with respect to camera lens distortion, position and angle using the Matlab Camera Calibration Toolbox (Computer Vision Research Group, California Institute of Technology).

Robot positions were used to produce an image (800×600 pixels) where uniform light discs of fixed blue intensity (Red = 0, Green = 0, Blue = 7) marked trail pheromone spots. Each disc was centered on the trajectory traced by a robot and did not overlap with the previous disc drawn along the same trajectory. Discs pertaining to different trajectories or that were not directly following each other on the same trajectory could overlap. In overlapping regions, pixel intensity corresponded to the sum over time of all the overlapping discs (up to a maximum blue intensity of 255). Finally, the light intensity (*I*) decreased following an exponential decay to simulate pheromone evaporation:
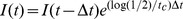
where 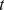 corresponded to the current time, 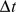 to the period between two evaporation time-steps and 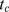 to the characteristic evaporation time (1800 sec). To lower the processing charge, evaporation was triggered every 5 seconds only 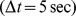. The tracking and trail laying software performed all computations at an effective speed of about 5 images per second. Given the robot speed of two body lengths per second and the maze dimensions this fulfilled our needs.

The final image was projected with a video-projector suspended about 3 m above the robots. Misalignment between the camera and the beamer was corrected using the Matlab Camera Calibration Toolbox. The projected image covered a surface of approximately 140×105 cm. The size of the blue disc after projection was fixed to 6 cm. This allowed the formation of light trails large enough for two robots to cross without being pushed outside the trail. These parameter values that produce consistent trail laying and trail following behaviors with these robots were established in a previous study [28].

### Behavioral model

The behavioral model was a generic model of trail laying and trail following behaviors in ants. Its purpose was to capture the essential features needed to achieve a path selection as it is observed in ant colonies [33]. In the absence of light pheromones, a robot (laying a trail or not) moved according to a correlated random walk, which is a random walk with a directional persistence, as is commonly found in insects [34]. This behavior is called “exploratory behavior”. If the robot detected an obstacle (with its built-in infrared detectors [31]), it tried to avoid it by turning away from the obstacle. This behavior was called “avoidance behavior.” If the robot detected a luminous trail with its photoreceptors, it tried to turn towards the brighter trail. This behavior was called “trail following behavior.”

Each of these behaviors triggered the computation of a movement vector. The exploratory vector 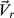 was a unit vector that initially points straight ahead of the robot and is modified at random time intervals. The new direction was chosen by drawing a random angle from a uniform distribution (using the Quick & Dirty algorithm [35]) between −30° and +30° and adding it to the current direction. The time intervals between each direction change were drawn from a decreasing exponential distribution with characteristic time being 3 seconds (i.e., an exponent of −1/3 second^−1^). Exponential random numbers were created from a uniform random number 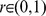 transformed to 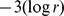 with an algorithm using only integers (see Ahrens and Dieter [36] for the algorithm).

The avoidance vector 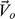 was the sum of four vectors (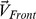, 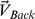, 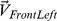, 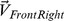), each of them pointing in the opposite direction of one of the four proximity IR sensors of the robot (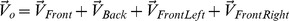). The intensity of each of the four vectors increased proportionally with the inverse of the distance between an obstacle and the corresponding sensor. Each sensor regularly and frequently emitted an IR signal that was reverberated by obstacles. The intensity of the reverberation perceived by the IR sensor was used as a proxy of the distance to the obstacle. This intensity diminished with the distance approximately following a sigmoid curve (0: the closest obstacle from the sensor is at least 3 cm away from it; 1: the obstacle is touching the sensor) [37].

The trail following vector 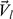 was the sum of two vectors pointing either to the right (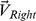) or to the left (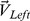) of the robots' current direction (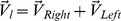). The intensity of 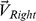 and 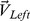 was controlled by the light intensities perceived by the right and left photoreceptor (0: no light perceived; 1: photoreceptor maximally stimulated).

The three vectors were summed together with different weights to obtain the direction 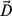 as a unit vector:
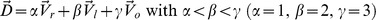



The robot then adjusted the direction and speed of the rotation of two independently driven wheels to achieve the new direction during the next step of its internal clock (50 ms).

Finally, the starting and the target areas in the experimental setup described above were equipped with two infrared transmitters that continuously emitted a signal. This signal was different for each area and the robots could detect it with their IR sensors. Each time a robot entered either the starting or the target area, it switched off its red LED, becoming invisible to the tracking software. As a consequence, it also stopped laying a light trail. This prevented robots from marking these areas while continuing their exploratory and obstacle avoidance walks. The red LED was switched on again as soon as the robot left the starting or the target area.

### Data collection and analysis

All data processing and statistical analysis were performed with R version 2.7.0 [38].

#### Individual behavior

In order to quantify the impact of the bifurcation structure on the individual displacement of the robots, we first analyzed their individual behavior when crossing an asymmetrical bifurcation. We tested whether their choice to follow the most direct branch resembled the choice observed in ant experiments [16].

During the first 2 minutes of each experimental replicate with network configuration A, we measured the proportion of robots crossing an asymmetric bifurcation and choosing to enter the most direct branch, i.e. the branch deviating by an angle of 30° from the original direction of the robot. Choices influenced by the contact with another robot at the bifurcation were excluded from this measure to be comparable to the individual choice data in [16] that had been obtained from isolated ants without direct interaction with a conspecific.

Deviation from a random choice was tested using a 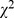 test for given probabilities. Comparisons with actual ant behavior at asymmetrical bifurcations (data taken from [16]) were performed with a Fisher's exact test for count data. Significant differences between robot and ant behaviors would suggest that the ants' choice is not simply dictated by the inertia of their movement.

#### Collective behavior

In order to quantify path selection efficiency in the different network configurations we had to assess (a) whether robots created a pheromone trail between source and target area and at what speed, (b) the length of this path and (c) the persistence of this trail. Efficient robots should find short paths quickly, short paths should persist longer than long paths, and there should be less changes in path selections.

We divided the network into segments, each of them corresponding to a corridor between two bifurcations. For each type of network, we computed the total pheromone intensity every second on each segment of the maze using data coming directly from the pheromone deposit device. This total intensity was obtained as the sum of all pixel values in one segment. A segment was considered as used when the total intensity was above a threshold of 50,000. This threshold roughly corresponds to the amount of light pheromone that 2 robots would deposit in a segment of the maze if they were following each other closely. Distributed over the entire segment, it also corresponds approximately to the minimum amount of light necessary to activate the trail-following behavior of a robot.

We determined the path selected by robots as follows. From the starting area, we followed the segment with the highest total pheromone intensity until we reached either a bifurcation followed by two empty segments (total intensity inferior to 50,000), a previously visited bifurcation or the target area. If we reached a bifurcation followed by two empty segments we counted this path in the “no path” category. If we reached a previously visited bifurcation we counted this path in the “loop” category. Finally, if we reached the target area we counted this path in a category named by its segment length. There were 7 different paths that connected the starting and the target areas without using the same segment twice, and these 7 paths belonged to 4 length categories: 4, 6, 8 and 10 segments.

**Figure 3:**
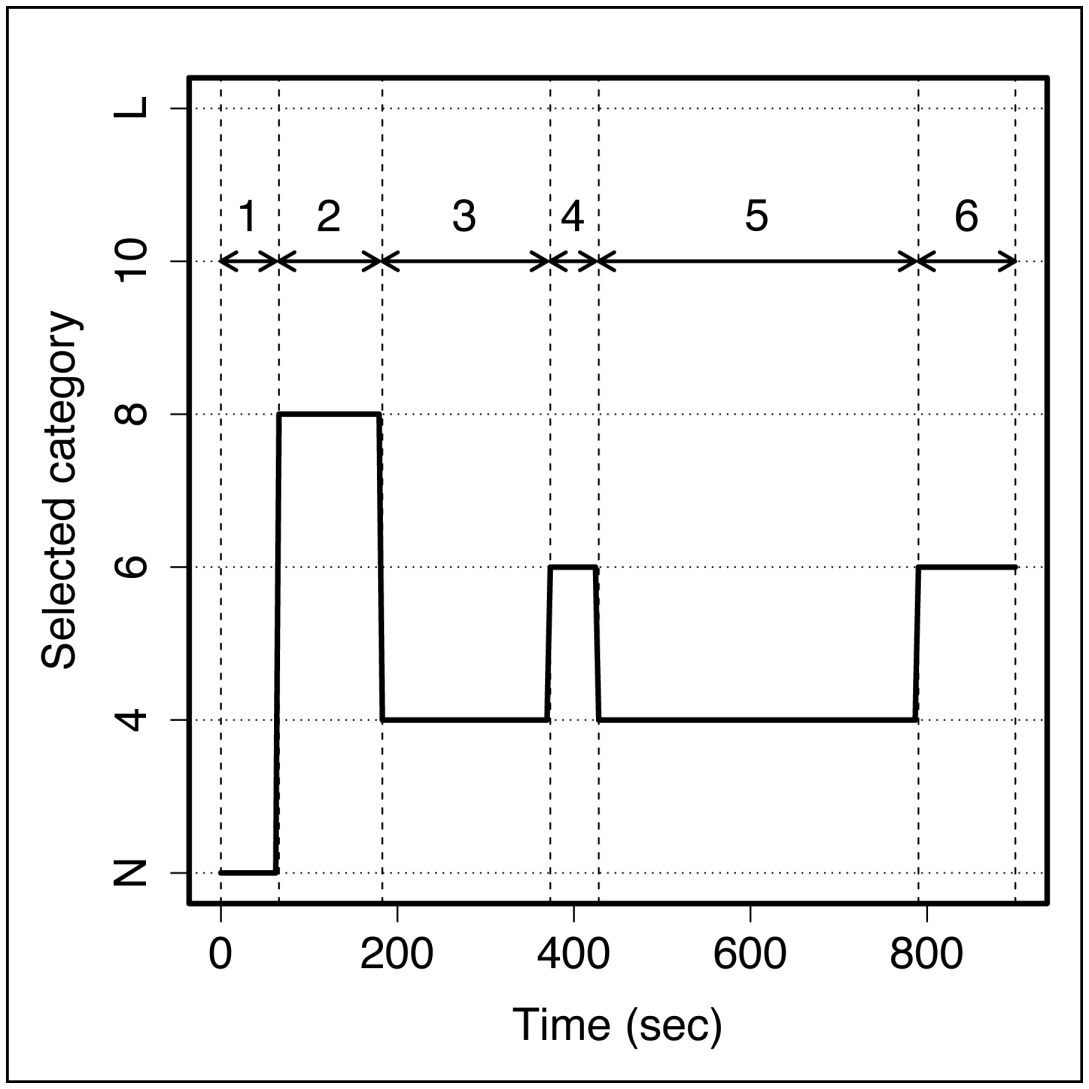
Example of a time sequence showing 6 different path selection events (numbered 1 to 6) at the beginning of an experiment. The x-axis represents the time from the beginning of the experiment. The y-axis represents the length of the path most used by the robots at a given time during the experiment. It is of 4, 6, 8 or 10 segments when the path is connected to the starting and the target areas; L when the path is connected to the starting or the target area only and forms a loop; N when the path is only connected to the starting or the target area and does not form a loop. In this example, there was no path selected during the first 60 seconds of the experiment (event 1), and then the group used a path with 8 segments until about 200 seconds (event 2), etc.

By repeating this process every second of each experimental replicate, we obtained the time sequences of path selection events (see Fig. 3 for an example). We grouped all consecutive frames that showed the same path category into a single event, called a selection event. We then computed the number of selection events and the durations of selection events for each selected path category for each experimental replicate.

Comparisons of the number of selection events between network configurations were performed using unilateral two sample Wilcoxon rank sum tests with continuity correction. Comparisons of the durations of selection events between path categories and between network configurations were performed using two-way ANOVAs and multiple pairwise comparisons were performed with a Tukey HSD test.

### Computer model

In order to investigate further the respective role of pheromone and network geometry on the overall foraging efficiency, we used a computer model of our system directly inspired from the one introduced in [16] for Argentine ants, but modified to account for the robots' specificities.

In the starting and target areas, robots perform a random walk (no pheromone) with obstacle avoidance. As a consequence their probability per unit of time of leaving the starting area, 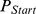, and the target area, 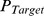, can be considered constant and equal (both areas have the same shape and dimensions). Once a robot has entered a segment 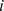 of the network, the time 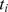 required to travel the segment is computed as follows: 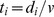, with 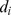 the length of the segment in centimeters and 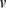 the speed of the robot (40 mm s^−1^).

At each symmetrical intersection, a robot has to choose between two segment 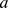 and 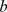. The probability 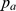 for an ant to choose the segment 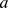 and 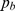 to choose the segment 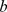 at a symmetrical bifurcation are modeled as follows:
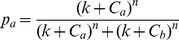


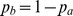
with 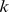 the intrinsic attractivity of segment 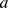 and 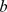, 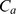 and 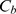 the quantity of pheromone on segment 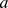 and 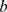, respectively, and 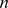 the degree of nonlinearity of the choice.

At an asymmetrical bifurcation, about 2/3 of the robots choose the segment deviating less from their incoming direction when the quantity of pheromone is equal on both segment. We computed the probability 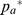 to select the segment 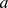 and 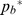 to select the segment 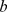 at an asymmetrical bifurcation as follows:
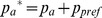


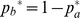


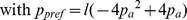


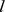
 corresponds to the tendency of a robot to move forward and chose the segment deviating less from its incoming direction. It is positive if segment 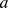 deviates by a 30° angle from the robot's incoming direction and negative if it deviates by a 120° angle. When 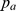 is equal to 0.5 (i.e., 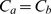), then 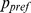 is equal to 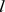, *i.e.* the robot's choice is influenced only by the geometry of the bifurcation because the two segments are equally marked with pheromone. Conversely, when one of the two segments becomes more marked with pheromone, then the robot's choice becomes influenced by the trail and we assume that the influence of the bifurcation geometry progressively decreases as the difference in pheromone concentration between the two segments increases. Therefore, when 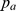 or 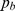 tend to 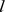 (*i.e.*, when 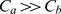 or 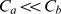), 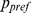 tends to 0.

Finally robots add a quantity of pheromone 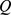 on each segment they visit. At each time step, the pheromone intensity (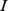) decreased following an exponential decay:
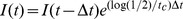
where 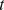 corresponded to the current time, 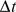 to the period between two time steps and 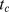 to the characteristic evaporation time (1800 sec).

A good match between the experimental data and the model is found for the following parameters values: 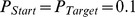; 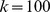; 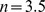; 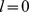 in configuration S, or 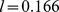 in configuration A; 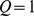 when pheromone deposition is allowed, or 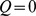 when it is not; 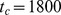.

## Results

### Individual choice at asymmetrical bifurcations

**Figure 4:**
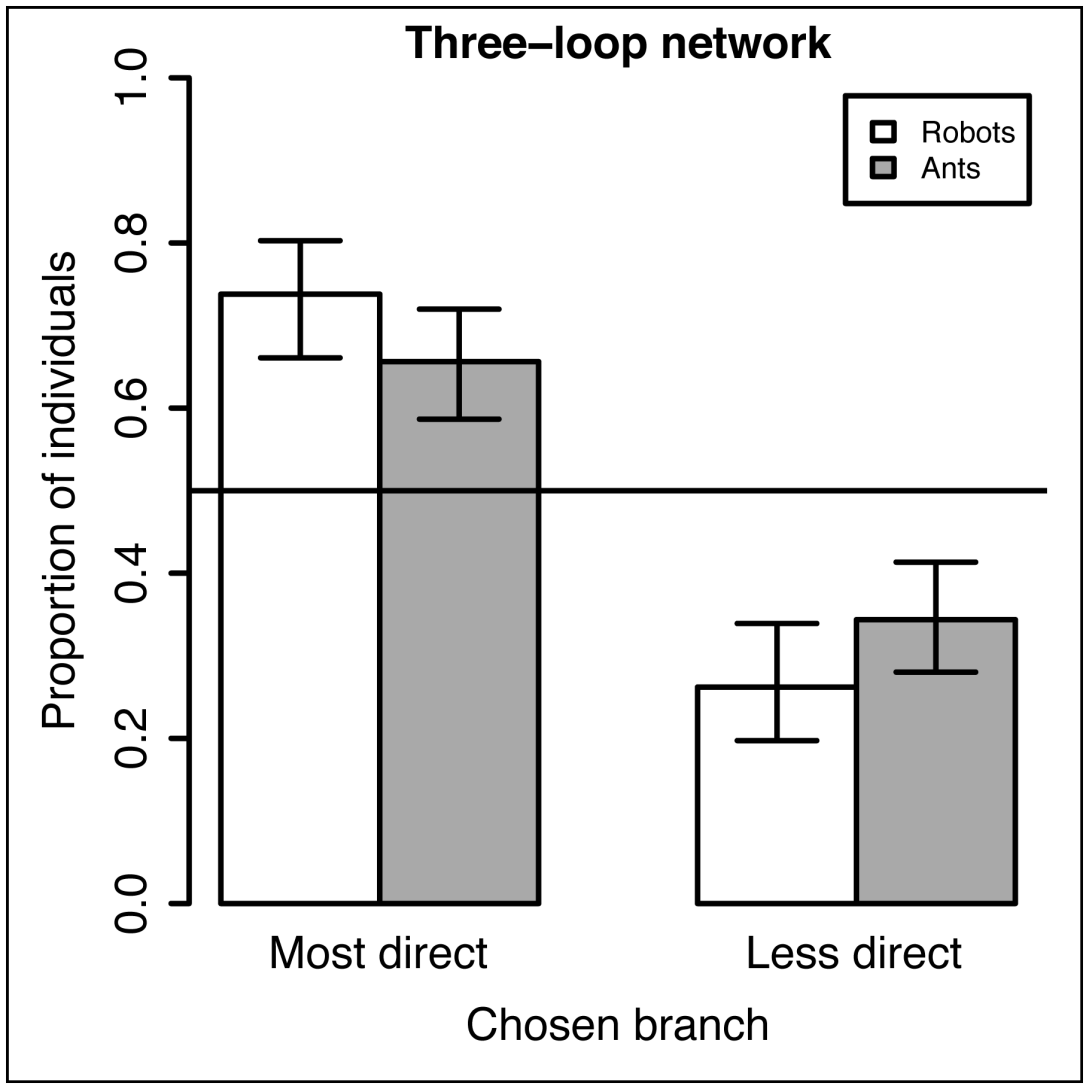
Comparison with the behavior of ants. In white, proportion of robots (with confidence intervals) selecting the most direct branch when reaching an asymmetrical bifurcation. In gray, proportion of ants selecting the most direct branch (data from [16]).

Results related to the individual behavior of ants (taken from [16]) and robots at asymmetrical bifurcations are summarized in Fig. 4. The figure shows how the proportion of individuals following a given branch is influenced by the angle this branch makes with the originating branch of the individual, in the absence of other information such as recruiting pheromone.

When reaching an asymmetrical bifurcation (configuration A, Fig. 4), both ants and robots chose more often to enter the branch deviating by an angle of 30° (126 observations in ants and 107 observations in robots) than the branch deviating by an angle of 120° (66 observations in ants, 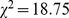, df = 1, p<0.001; 38 observations in robots, 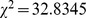, df = 1, p<0.001). Additionally, the proportion of robots entering the most direct branch was not significantly different from the one observed in ants (107/145 = 74% for robots vs. 126/192 = 66% for ants, Fisher's exact test, p = 0.122).

As shown by these results, the choice behavior of the robots at an asymmetrical bifurcation is similar to the one of the ants. In this initial phase of the experiment, branches do not bear yet any pheromone marking but the robot's simple correlated random walk leads them to “choose” the branch that deviate less from their current trajectory. This shows that no complex orientation strategy is required to reproduce the individual choice behavior of the ants with the robots.

### Collective choice in symmetrical and asymmetrical networks

**Figure 5:**
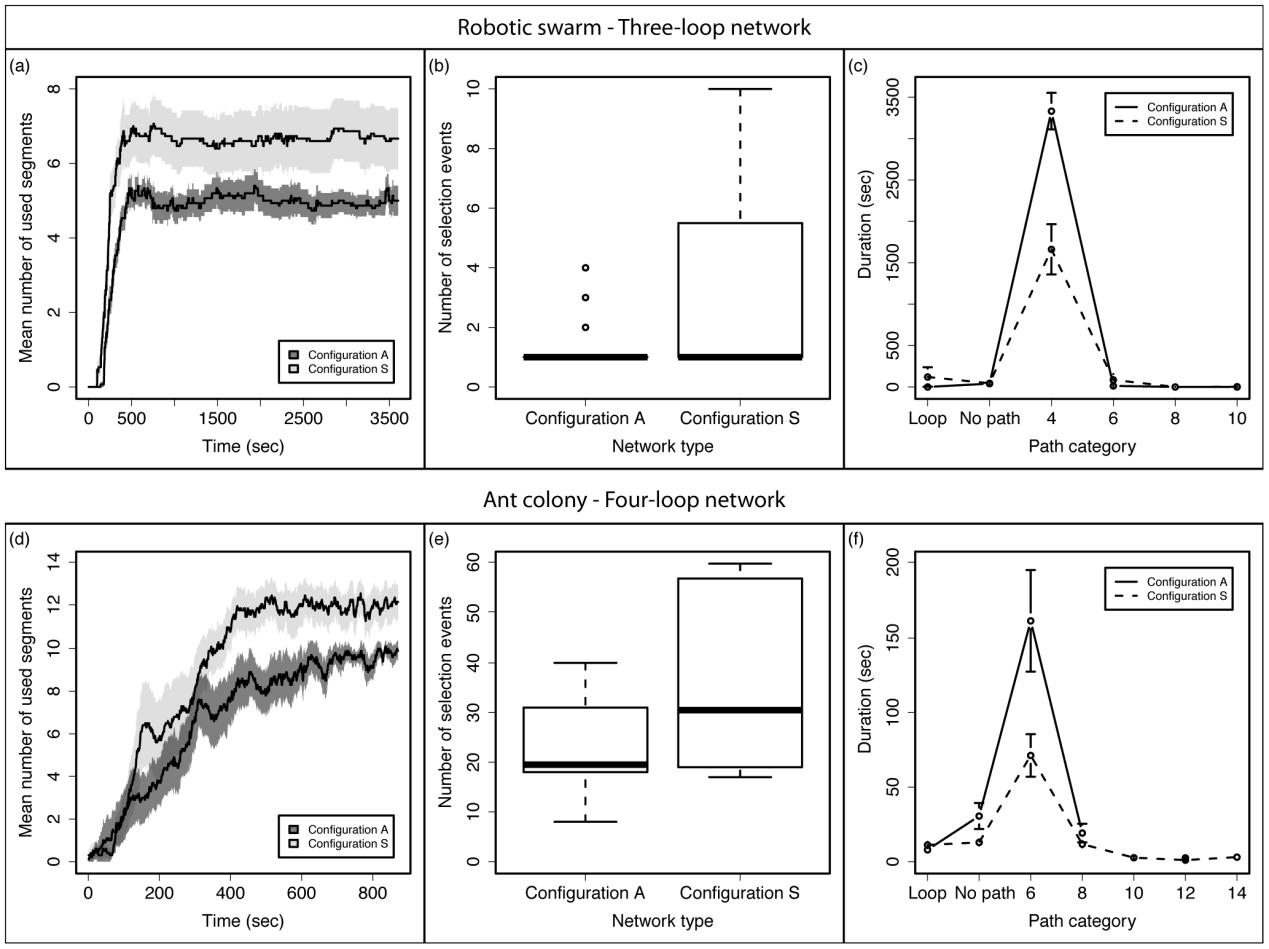
Collective path selection results for the three-loop networks. The top row (a–c) corresponds to the robotics experiments (15 experimental replicates with each network configuration) with a three-loop network presented in this article. The bottom row (d–f) corresponds to the experiments (10 experimental replicates with each network configuration) performed with ant colonies in [16] with a four-loop network and is reproduced here for purpose of qualitative comparison. (a & d) Number of network segments used by robots/ants as a function of time. The black line represents the mean value with its standard error area band (light gray for configuration S, dark gray for configuration A). Note the different time scales between robot and ant experiments. (b & e) Number of selection events observed over the course of the experiments. Each bar represents the boxplot for each network configuration. (c & f) Duration of selection events by path category and by network configuration. The dashed line represents mean values (+/− sem) obtained with the network in configuration S, the continuous line represents mean values (+/− sem) obtained with the network in configuration A. On the x axis, numbers represent the length of the selected path.

Results from the collective path selection experiments are summarized in Fig. 5. The typical time course of an experimental replicate is shown in Fig. 2c and Video S1. As observed in ants (see [16]), robots dispersed in the network during the first minutes of the experimental replicate, before limiting their displacement to a single path connecting the starting and the target areas. This path was the shortest possible path at the end of all 15 experimental replicates in both configurations A and S.

The number of network segments used by the robots increased rapidly during the first 500 seconds of an experimental replicate (see Fig. 5a), which corresponded to the initial dispersion of the individuals inside the maze. It reached a plateau value around which it oscillated during the rest of the experimental replicate. This plateau value was different between the two configurations, with a mean number of segments used at around 7 for configuration S and around 5 for configuration A. While the ant and robot experiments differed in population and maze size, the dynamics of the number of segments used in both cases were qualitatively similar (see Fig. 5a vs. d) and indicated a more important dispersion of the individuals in configuration S of the network. Although both ants and robots tend to find the shortest path in both configurations, there is more dispersion away from this path in configuration S.

In order to determine if the robots preferentially used one particular path category, we computed the mean duration of the observed selection events for each path category, which is the mean time during which the robot colony preferentially used a path category before switching to another path category (see Fig. 5c). This duration varied significantly among the different path categories (see Fig. 5c, 2 way ANOVA, F = 29.27, df = (4,94), p<0.001), with the shortest path category being selected for the longest time in both network configurations (Tukey HSD, p<0.001 when comparing the 4-segment category with the other path categories; comparisons with 8- and 10-segment categories was not possible since they were selected respectively 1 and 0 times only during all the experimental replicates). Moreover, the mean duration of observed selection events was significantly longer in configuration A (2 way ANOVA, F = 10.31, df = (1,94), p = 0.002), as was the mean duration of selection events for the shortest path category (Tukey HSD, p<0.001). These results are qualitatively similar to those observed in ants (see Fig. 5c vs f) that also preferentially used the shorter path category in both configurations of the network, and used it more consistently in configuration A than in configuration S.

The previous observation was corroborated by the analysis of the number of switches between the different path categories during an experimental replicate. Robots that started using one path category could switch to another one several times during an experimental replicate, but the number of observed selection events was significantly smaller when the network was in configuration A than when it was in configuration S (see Fig. 5b, W = 72, p = 0.024). A similar result was also observed in ants (see Fig. 5b vs. e and [16] for its statistical analysis).

### Respective role of pheromone and network geometry

We ran the computer model under four different conditions - configuration S with and without pheromone deposition, and configuration A with and without pheromone deposition - and we compared the ability of the robotic group to complete successful trips between the starting and target areas.

**Figure 6:**
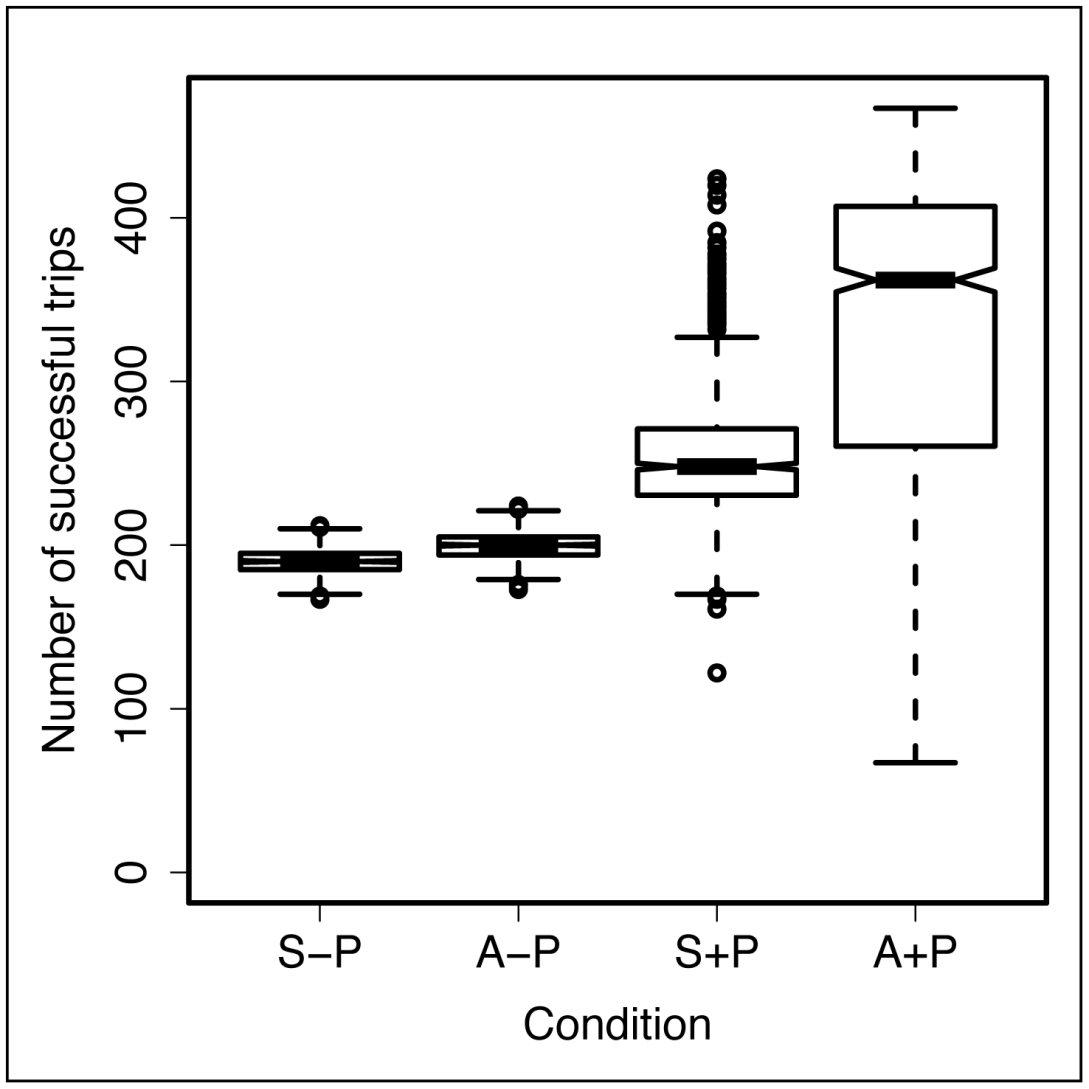
Foraging efficiency of the robots as given by simulations of our model. The foraging efficiency is measured as the number of successful trips, i.e. the number of times a robot has returned to the starting area after visiting the target area. Each boxplot represents the values for 1000 simulation runs. The black horizontal bar in each boxplot represents the median; the notches around the median represent the confidence interval of the median. The four tested conditions were: configuration S without pheromone deposition (S-P); configuration A without pheromone deposition (A-P); configuration S with pheromone deposition (S+P); configuration A with pheromone deposition (A+P).

For each of the four conditions, we ran 1000 simulation runs. The foraging efficiency of the robotic group under each condition is summarized in Fig. 6. The foraging efficiency is expressed as the number of successful trips performed by the robots, *i.e.* the number of times a robot has returned to the starting area after visiting the target area.

In absence of pheromone (S-NP and A-NP in Fig. 6), robots placed in a network with configuration A performed significantly better than those placed in a network with configuration S (Wilcoxon rank sum test, W = 193972, p<0.0001). However the amplitude of the improvement was small: the robots in configuration A completed only 1.05 times more successful trips than those in configuration S (measured as the ratio between the median number of successful trips in both conditions).

The addition of pheromone in the model led to a significant increase in the number of successful trips when the robots were in configuration S (W = 16538.5, p<0.0001) and configuration A (W = 171869, p<0.0001). In configuration S, robots completed 1.3 times more successful trips when pheromone was added to the model than when it was absent. This ratio grows to 1.8 in configuration A. Finally robots completed 1.46 times more successful trips in configuration A than in configuration S in the presence of pheromone.

In conclusion our simulations show that the geometry of the network has an influence on the foraging efficiency of the robots, but this influence is small compared to the one of the pheromone (compare 1.05 with 1.3). When combined they result in a nonlinear increase in the foraging efficiency (compare 1.05 and 1.3 with 1.8).

## Discussion

In numerous ant species, pheromone trails play an essential role during foraging tasks by guiding workers toward previously discovered resources or helping them finding their way back to their nest [1]. In certain species these trails form an intricate network, thus challenging the navigation abilities of ants [3]. Recent studies have shown that the geometrical structure of the trail network directly affects the choice of which path to follow when an ant crosses a bifurcation, and thus modifies the foraging efficiency of the colony [14]–[16]. It was less clear however whether or not individual workers were actively considering the geometry of a bifurcation when choosing a path to follow, though this feature was used in previous simulation work [16], [17].

Using a robotic model, we have shown that no representation or even simple detection of the presence of a bifurcation was necessary to explain the individual ant behavior. The robots were not explicitly programmed to identify the presence of a bifurcation or to estimate its geometrical configuration. Instead they were programmed only to move according to a correlated random walk and to avoid obstacles indifferently of their nature, be they gallery walls or other robots. Yet their behavior when crossing a symmetrical or an asymmetrical bifurcation was comparable to the behavior of Argentine ants in similar situations, suggesting that the individual decisions of Argentine ants at bifurcations are affected by the physical structure of the environment in a passive way (*i.e.*, without the formation of a representation of the bifurcation prior to the decision). Considering the poor performance of the Argentine ants' visual system [39] and the high tempo of the workers along the trail (up to 2.5 cm s^−1^ for an average body length of 3 mm, personal observation), it is unlikely that Argentine ants would have the time and capacity to evaluate the geometry of a bifurcation that they would cross in less than half of a second (the length of a bifurcation in [15], [16] is about 1 cm from the entrance to one of the two possible exits). Our results show that such a complex cognitive process is not necessary to explain the ants' behavior.

At the collective level, the interaction between the pheromone-based recruitment process and the tendency to move into the least deviating branch of the bifurcation created a significant difference in the pattern of network use between symmetrical and asymmetrical networks. While the robots tended to more intensely use the shorter path between the starting and target areas in both configurations, robots collectively more consistently selected the shorter path and tended to spread less in the asymmetrical network. This result was qualitatively very similar to what was observed in ants, though a quantitative comparison was not possible because of the large-scale differences between the two systems (differences in relative speed or quantity of pheromone deposited for instance). Experiments with ants were also performed with colonies of 500 workers [16], while only 10 robots were used in each of our experiments. This resulted in a larger dispersion of the individuals in the ant experiments as shown by the greater number of selection events (see Fig. 5e). This increased dispersion is probably caused by overcrowding on the trail that favors the use of alternative routes [28], [40]. Argentine ants are also known to perform more U-turns with increasing deviations from their initial trajectories [15], [16]. However this behavior does not seem to affect the collective ability of the colony to select the shortest path in the network, as shown by simulations in [16]. Our results support this observation as robots in our experiments were not explicitly programmed to perform U-turns (though collisions with other robots can lead to such U-turns) and yet their collective behavior was similar to that of ants. Note that pheromone marking is essential for the path selection to occur. Without pheromones, robots would simply diffuse in the network according to their correlated random walk and approximately reflective obstacle avoidance behavior. Assuming quasi-instantaneous direction changes (relative to the moving speed of the robots, rotation time is negligible here), standard diffusion theory [41] predicts a completely homogeneous distribution of the robots in the network at stationary state (reached in our system within 10 minutes, see Fig. 5a). Even moderate deviations from these assumptions could not lead to the preferential use of the shorter path by the robots.

Finding the shortest path between two nodes in a network requires solving a series of binary choices at each bifurcation. Following the wrong path at one bifurcation can propagate over the following decisions because of the persistent nature of the attractive pheromone, therefore decreasing the chances of finding the best solution, or even locking the system in a loop. This study shows that the coupling of a particular geometrical configuration of trail networks and the forward oriented movement of ants reduces the chances of a bad choice and favors the selection of one of the shorter paths between the nest and the food source. It has an effect similar to the heuristic information in Ant Colony Optimization (ACO) algorithms [42]–[44]. Both provide a general axis for the information to propagate and therefore reduce the probability that ants (virtual and natural) get trapped in loops or less efficient solutions [44], [45].

This last remark raises the question of the origin of the particular geometry of the trail networks built by several ant species. In their work about foraging trails in the ant *L. processionalis*, Ganeshaiah and Veena (see [12] and references therein) mention that a branching pattern is a good trade-off in minimizing both the total length of the network and the average distance between two endpoints (where food can be localized for instance). They also note that bifurcation angles that minimize the resistance to the movement of the ants in such networks should be around 70°–80°, which is close to what has been found afterward in several ant species [3], [13], [14]. This last point suggests that the formation of a bifurcation may be strongly influenced by the movement of ants along a trail, and that the formation of specific geometrical configurations may not require complex cognitive abilities. One possible scenario to explain the emergence of these particular angle values could be the following. A first phase of random exploration around the nest or the endpoint of an existing trail would result in a random network of weak trails. Then the passage of ants along these trails combined with their forward oriented walk would reinforce bifurcation branches that deviate from the originating direction of the ants by no more than a threshold angle (possibly 30°–40° from the originating direction of the ant, i.e. an angle of 60°–80° between the two branches). Largely deviating branches would be therefore abandoned little by little. Furthermore, at bifurcations where the branches would be very close to each other, the natural diffusion of the pheromone and its imperfect detection by ants would eventually lead to the fusion of the two branches into one trail only, thus preventing the maintenance of smaller angles between the two branches of a bifurcation. A recent model of trail formation introduced in [46] confirms part of this scenario.

Finally, our findings emphasize the interplay between the behavior of a swarm system and the configuration of the environment into which the swarm system moves. While most studies of ant-made networks focus on the efficiency of their topological properties (see for instance [3], [47], [48]), we show here that their geometrical configurations also affect the spatial distribution of individuals, and hence the foraging efficiency of the colony [16]. On a related note, Batty [49] suggested that the configuration of a building could explain why a human crowd would favor certain spaces and routes more than others. We also suspect that within an ant nest, local geometrical constraints might favor the formation of preferred paths channeling the motion of ant workers. Similarly, several swarm robotics studies have shown that the shape of interacting robots could be responsible for the emergence of collective patterns [50]–[52]. In all these cases, the physical configuration of the environment (the structure of the network, the organization of the rooms or the shape of the other individuals) directly influences the collective outcome and can potentially modify the pattern of interaction and information exchange between individuals. Understanding the constraints applied by the environment on the behavior of individuals should make it possible to use them appropriately to improve the design of crowded areas or to favor the emergence of certain desirable behaviors in a swarm of robots.

## Supporting Information

Video S1 Typical time course of an experiment (1 hour). The starting area is in the top right corner, the target area in the bottom left corner.
(MP4)
